# Risk Factors Associated with Esophageal Fistula after Radiotherapy for Esophageal Squamous Cell Carcinoma

**DOI:** 10.7150/jca.39033

**Published:** 2020-03-31

**Authors:** Bing Hu, Feng Jia, Haiyan Zhou, Tao Zhou, Qian Zhao, Yiru Chen, Baosheng Li, Wei Huang

**Affiliations:** 1 School of Medicine and Life Sciences, University of Jinan-Shandong Academy of Medical Sciences, Shandong province, China; 2 Department of Oncology, Jinxiang people's hospital, Shandong province, China; 3 Department of Medical Oncology, Shandong Cancer Hospital and Institute, Shandong First Medical University and Shandong Academy of Medical Sciences, Shandong province, China; 4 Department of Radiation Oncology, Shandong Cancer Hospital and Institute, Shandong First Medical University and Shandong Academy of Medical Sciences, Shandong province, China

**Keywords:** esophageal squamous cell carcinoma, esophageal fistula, radiotherapy, risk factor, side effect

## Abstract

**Purpose:** The aim of this study was to investigate risk factors for esophageal squamous cell carcinoma (ESCC) treated with radiotherapy (RT) with or without chemotherapy to guide how to reduce the occurrence of esophageal fistula (EF).

**Methods:** 414 patients with ESCC who underwent RT with or without chemotherapy were collected in Shandong Cancer Hospital from February 2012 to June 2018 retrospectively. The clinical characters and dosimetric parameters were recorded. Univariate and multivariate logistic regression analyses were provided to determine the risk factors associated with EF.

**Results:** The cumulative incidences of EF were 11.1% (46/414 patients). The median follow-up period was 15.8 months (range: 0.2-82.6months). The median survival time (MST) of patients with EF was 5.3 months. In univariate analysis, age, T4 stage, N3 stage, chemotherapy regimens, re-RT, ulcerative esophageal cancer (EC), esophageal stenosis, the maximum thickness of the tumor and the length of tumor had a correlation with the prevalence of EF. In multivariable logistic regression analysis, T4 stage, N3 stage, re-RT, ulcerative EC, esophageal stenosis, the maximum thickness of the tumor was confirmed as risk factors for EF.

**Conclusion:** This study revealed that T4 stage, N3 stage, re-RT, ulcerative EC, esophageal stenosis, the maximum thickness of the tumor were risk factors associated with EF. We ought to attach importance to the prevention of EF. Patients with risk factors for EF should be paid close attention.

## Introduction

About half of newly diagnosed esophageal cancer (EC) cases occur in China each year. The incidence of EC in China ranks third in malignant tumors, and the mortality rate ranks fourth 1. More than 90% of patients with EC have pathological type of esophageal squamous cell carcinoma (ESCC). About 40%-50% patients have lost the chance of surgery for advanced ESCC when they were first diagnosed. Chemoradiotherapy (CRT) is the standard for unresectable ESCC 2-7. CRT can improve the survival rate of patients with ESCC 8. However, side effects of CRT might also occur, especially esophageal fistula (EF), which is a serious complication. Anatomically, the esophagus is surrounded by the trachea, lungs, aorta, mediastinum and pericardium, which are often invaded by advanced EC. On the one hand, CRT can induce EF which by damaging the walls of the esophagus and adjacent organs. On the other hand, the imbalance between tumor shrinkage and normal tissue repair can lead to EF 9-11. Although the incidence of EF is low (10.4%-13.9%) but the prognosis is poor and the death rate is high 12,13. EF directly reduces the quality of life of patients and affects the therapeutic effect. EF can easily lead to serious and life-threatening infections. Most patients with EF die in 3-4 months due to infection and malnutrition 14,15. Therefore, early prevention of EF to reduce the incidence is very important. Although EF is critical for these patients, the associated risk factors have not been clarified. We undertook this study to answer this question.

## Materials and Methods

### Patients' selection

419 ESCC patients who were treated with RT in Shandong Cancer Hospital were collected from February 2012 to June 2018 in this study retrospectively. 5 patients who were lost to follow-up were excluded. All data were collected from electronic medical records. The study was approved by the Ethics Committee of the Shandong Cancer Hospital and Institute (SDTHEC20171208). We confirmed that all research was performed in accordance with relevant guidelines. We confirmed that regulations and informed consent was obtained from all participants and their legal guardians. The inclusion criteria we followed are: 1. All patients who had pathologically confirmed ESCC; 2. Staged as II-IV based on the American Joint Committee on Cancer (6th edition); 3. Treated by RT or re-RT with or without chemotherapy; 4. Karnofsky performance status (KPS) score≥70; 5. Patients without any other serious medical illness except EC; 6. No EF before RT. The exclusion criteria we followed are: 1. The patients underwent esophageal surgery previously; 2. Lost to follow-up.

### Data collection

The following clinical characters and dosimetric parameters were recorded and analyzed. Clinical characters include age, gender, KPS, smoking history, drinking history, location of the tumor and stage, the length of tumor, the maximum thickness of the tumor, ulcerative EC, esophageal stenosis, treatment modalities, chemotherapy regimens, cycles of chemotherapy. Dosimetric parameters include fraction dose, total radiation dose, re-RT, RT field.

### Pretreatment evaluation

All patients underwent a physical examination, pathological and cytological examination by esophagoscopy, contrast-enhanced Computed Tomography (CT) of the cervical and chest region, endoscopy of the esophagus, endoscopic ultrasonography, barium esophagography. The T stage was diagnosed by surgeons, oncologists and radiologists based on findings of endoscopic ultrasonography and enhanced CT. In many advanced patients, endoscopic ultrasonography was optional because the esophagoscope could not be passed through stenotic lesions. The maximum thickness of the tumor was measured on CT, magnetic resonance imaging (MRI) or Positron Emission Tomography-Computer Tomography (PET-CT) by taking the maximum thickness of internal diameter and external diameter. The tumor length was defined by endoscopy of the esophagus, barium meal, CT, MRI, or/and PET-CT. Esophageal stenosis was determined according to the patient's symptoms and the narrowest transverse diameter in a barium meal examination. All patients were evaluated at 3 months intervals for the first 2 years, and at every 6 months thereafter. At each visit, evaluation consisted of physical examination and medical history, new symptoms were also recorded, a enhanced CT scan or a barium esophagography was performed to check for EF.

### Treatment programs

All patients with ESCC included in the study were treated with concurrent CRT, sequential CRT or RT alone.

### Chemotherapy

Patients with ESCC generally chose the following two chemotherapy regimens: DP scheme included docetaxel (TXT) 75 mg/m2/day or paclitaxel 135-150 mg /on days 1, and cisplatin (DDP) 25 mg/ m2/day on days 1-3. PF scheme include 5-fluorouracil (5-FU) 1000 mg/ m2/day on days 1-5 or S-1 60-80mg/ d1-14 and DDP 25 mg/ m2/day on day 1-3. The above schedule was repeated every 21-28 days.

### Radiotherapy

Each patient was placed in the supine position with a body vacuum bag or head and neck thermoplastics. The scanning range was from the ring membrane to the 5 cm below the lower edge of the lungs. A slice thickness of 3.0 mm. CT image was transmitted to the Varian Eclipse 8.6.15 planning system for delineation and planning of the target area and the endangered organ. Gross tumor volume (GTV), Clinical target volume (CTV) and Planned target volume (PTV) were delineated on the CT image. GTV was the range of tumors and metastatic lymph nodes that could be seen on CT/PET-CT/MRI. CTV includes GTV subclinical lesions and high-risk lymphatic drainage areas 16. The PTV was defined as 0.5-0.8 cm beyond the CTV. Radiation was administered via a 6 MV X-ray. Most RT doses were 1.8-2.5 Gy (five times a week). Maximum dose of radiation from the spinal cord ≤45Gy, the mean doses of the heart were ≤30 Gy, the volume of the lung receiving 20 Gy (V20) ≤33%.

### Definition of EF

**Figure 1:**
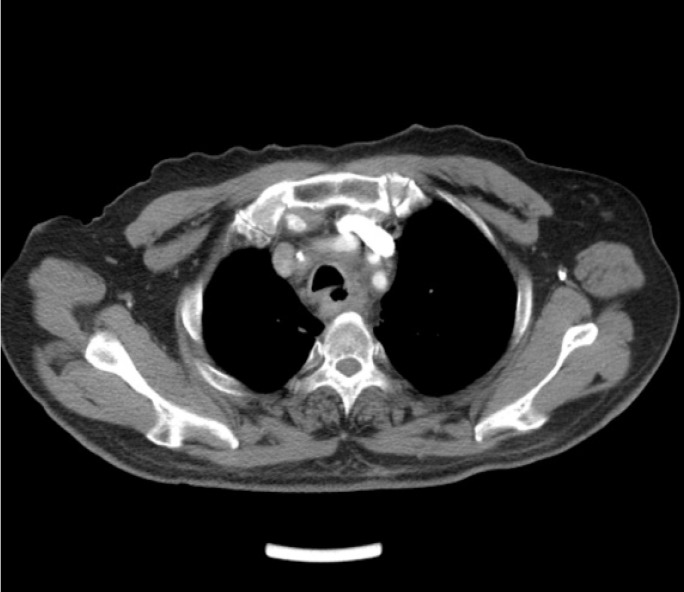
Axial contrast-enhanced computed tomographic (CT) scan of the chest shows esophagorespiratory fistula.

**Figure 2:**
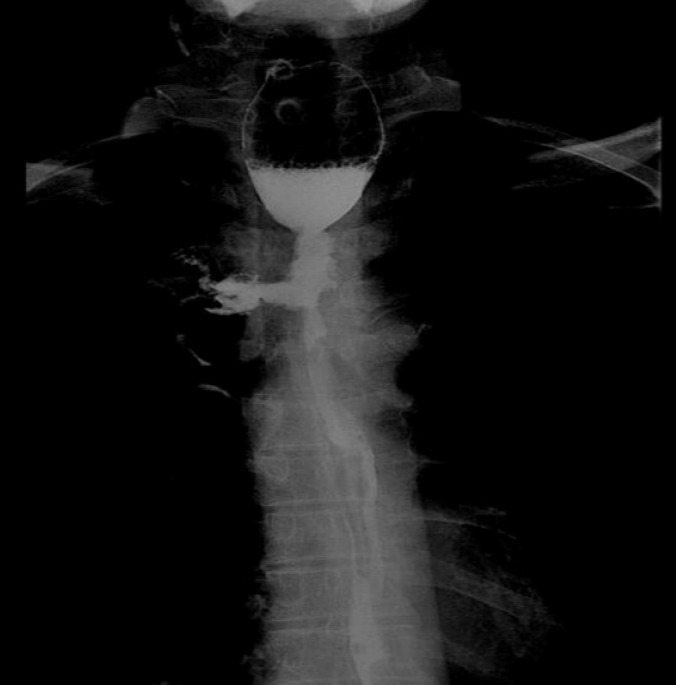
Barium esophagography shows esophageal-mediastinum fistula.

EF was a connection between the esophagus and adjacent organs 17. The diagnosis of EF was routinely confirmed by cervical and chest CT, barium esophagography or endoscopy of the esophagus during RT or after RT. The common clinical manifestations of EF include dramatic cough with massive sputum or hematemesis, chest pain and fever. Types of EF include EMF (Esophageal-mediastinum fistula), ERF (Esophagorespiratory fistula) and AEF (Arterio-esophageal fistula). Typical imaging of EF seen in Fig 1-2.

### Statistical analysis

Data of all patients were summarized and analyzed retrospectively. The incidence of EF during or after RT was calculated for all patients. Univariate analysis was performed for 21 variables by logistic regression methods. For the multivariate analysis, logistic regression was used for the selection of informative risk factors. Univariate and multivariate analyses were carried out using logistic regression to estimate the odds ratio (OR) and 95% confidence intervals (CIs). Differences with p-values <0.05 were considered statistically significant. All analyses were performed using IBM SPSS Statistics version 25.

## Results

### Patient characteristics

414 patients were included in the analysis. There were 319 males (77.05%) and 95 females (22.95%) . EF was observed in 46 patients (40 males and 6 females), and the incidence of EF was 11.11%. The median age was 65 years (range 32-88 years), 46 patients in the cervical section (11.11%), 123 patients in the upper thoracic location (29.71%), 192 patients in the middle thoracic location (46.38%), and 53 patients in the lower thoracic location (12.8%). 155 patients with T4 stage ESCC (37.44%). 154 patients have ulcerative ESCC (37.2%). 340 patients had esophageal stenosis (82.13%). The median length of tumor was 5 cm (range 0.7-17.06). The median tumor thickness was 15.31 mm (range 4-41.21). 98 patients received only RT (23.67%), 120 patients received concurrent CRT (28.99%) and 196 patients received sequential CRT (47.34%). DP chemotherapy regimens was used in 191 patients and PF in 125 patients.

The types of EF in this study included 30 patients with ERF, 16 patients with EMF. EF occurred in 8 patients during RT, and in 38 patients after RT. The patient characteristics were listed in Table 1.

### Survival

All follow-up data were updated at the end of December 2018, resulting in a median follow-up period of 15.8 months (range: 0.2-82.6months). During the treatment of all enrolled EC patients, the clinical manifestations of EF should be closely observed. The median survival time (MST) of patients with non-EF was 36.8 months. The 1-year survival rate of the patients was 27.1%, and the 2-year survival rate was 13.9%. The median time interval between the date of RT completion and the date of EF diagnosis was 2.4 months (range: 0-19.3months). The average time of EF was about 3 months after RT. The MST of patients with EF was 5.3 months. The Kaplan-Meier method was used to calculate the survival time from the first day of RT to the day of death or the last day of confirmed survival. The prognosis of patients with EC after EF was very poor. The research results showed that 13 patients (28%) died within 3 months after the diagnosis of EF, and 26 patients (57%) died within 6 months of EF. Because of the different nature of EF, patients had different survival times. The MST of ERF was 5.3 months and that of EMF was 4.65 months (P=0.991). The survival rate of patients with EF by December 2018 was 24%. Overall survival curves were estimated using the Kaplan-Meier method.

### Risk Factors for EF

Among the tested 21 variables including age, gender, smoking history, drinking history, T4 stage, N3 stage, M1 stage, TNM clinical stage, location of primary tumor, KPS, the length of tumor, the maximum thickness of the tumor, ulcerative EC, esophageal stenosis, treatment modalities, chemotherapy regimens, cycles of chemotherapy, fraction dose, total radiation dose, re-RT, RT field. Table 2 shows the results of univariate analyses of the risk factors for EF. The meaningful factors were included in multivariate analysis. T4 stage, N3 stage, re-RT, ulcerative EC, esophageal stenosis, the maximum thickness of the tumor had a significant correlation with the prevalence of EF. The detailed information was shown in Table 3.

## Discussions

EF is a devastating and life-threating complication. It can cause pneumonia, lung abscess, sepsis and even death. Once EF occurs, the prognosis is extremely poor. The average survival time of EF is 2-3.2 months 13,17-19. In this study, the MST of patients with EF was 5.3 months. Early prevention of EF is very important. The risk factors associated with EF in patients receiving RT have not been elucidated. Therefore, we conducted this study to determine the relevant risk factors. EF was the result of tumor necrosis and mucosal injury caused by RT 20. Radiation therapy could cause rapid necrosis of tumor tissue, fibrosis of the esophageal wall, and poor local blood supply. If the normal tissue could not be repaired in time, EF might occur 9. 21 clinical and dosimetric factors were included in the study. T4 stage, N3 stage, re-RT, ulcerative EC, esophageal stenosis, the maximum thickness of the tumor were risk factors for EF.

ESCC invading surrounding tissues and adjacent organs is related to the high incidence of EF 21. Stage T4 tumors invaded the entire layer and adjacent structures of the esophagus, so they could not be surgically removed. CRT were the standard treatment. Patients with T4 stage EC was more likely to develop EF after CRT, with an incidence of 18-29% 21,22-27. Among the 46 patients with EF in the present study, the number of patients in stage T4 was 1.42 times higher than in those with non-T4 stage. The patients with higher N stage of EC tend to have more extensive lymph node metastasis and the larger RT field, which may easily lead to EF. We also found that the larger maximum thickness of the tumor was prone to EF. This might be due to the imbalance between tumor tissue contraction and normal tissue repair system 9,10.

Patients with esophageal stenosis had a significantly increased risk of EF with CRT 28. We graded the degree of esophageal stenosis by barium esophagography examination. It was found that EF was more likely to occur when the esophageal diameter was <0.5 cm. Of the 46 patients with EF, 41 patients had esophageal stenosis (89.13%). The incidence rate of EF for patients with stenosis was 12.06%, and 6.33% for those without stenosis. Tsushima et al.^28^ revealed that esophageal stenosis was the only risk factor for EF. The cause of this phenomenon had not yet been clarified. This might be due to the friction of food on the esophagus.

The study found that radiation dose was not associated with the occurrence of EF. An important reason might be that 70% of patients had a radiation dose of 60±7Gy. There was no significant difference in the patient's radiation dose. Re-RT was a strong risk factor for EF (P=0.003, OR=3.926 ,95%CI: 1.615-9.548). Zhou et al.^29^ reported 55 patients with recurrent EC happened EF after re-RT. The study found that re-RT could improve survival rate of patients, but the incidence of EF was as high as 20% (11/55). Kim et al.^30^ retrospectively analyzed that 17 patients with recurrent EC were treated with re-RT after primary RT. EF occurred in 3 patients (17.6%). The incidence of EF in re-RT was significantly higher than primary RT. Therefore, for patients with re-RT, more attention should be paid to prevent the occurrence of EF. It is not clear that the suitable re-RT dose for patients with recurrent EC. Next, we will continue to study the risk factors of EF in patients with ESCC receiving re-RT.

Ulcerative EC often reached the muscular layer or penetrates the muscular layer. EF might occur in patients with increased pressure in the lumen due to swallowing or severe cough. In addition, local ulcer lesions were often accompanied by infection, which increased the chance of EF. Sun et al.^31^ found that 17 patients developed EF, 11 patients were deep ulcerative EC (64.7%). Tsushima et al.^28^ set a study confirmed that 89% of patients with EF had ulcerative EC. Among the patients included in the study, patients with esophageal ulcer account for 54.35% of the total number of patients with EF, indicating that patients with ulcerative EC were prone to EF. Therefore, clinical doctors should be cautious of RT for patients with esophageal ulcers.

Our study showed that T4 stage, N3 stage, re-RT, ulcerative EC, esophageal stenosis, the maximum thickness of the tumor were risk factors for EF. The findings had significant meaning. Patients with high risk factors of EF should be cautious about RT. Some previous reports showed that patients had received induction chemotherapy before CRT reduces the incidence of EF 32,33. Therefore, induction chemotherapy followed by CRT may be a feasible treatment for the patients with risk factors. This might be because induction chemotherapy can cure early EF or deep ulcers.

During the course of RT, patients with chest pain, cough and fever could be diagnosed by barium esophagography and CT of the cervical and chest region as early as possible to determine whether EF had occurred. Patients with advanced EC had long-term dysphagia and malnutrition, leading to poor repair ability of normal tissue, which was easy to cause EF. We should let patients to strengthen nutrition and treat anemia to reduce the incidence of EF. Once EF occurs, it is necessary to diagnose and treat as soon as possible to improve the quality of life and prolong survival. We believe these results can be verified in future prospective surveys.

The present study had several limitations. First, this was a retrospective study from one institution. Second, it was difficult to distinguish whether EF was caused by RT or disease progression. Third, no patient developed esophageal aortic fistula in this study.

## Conclusion

This study showed that T4 stage, N3 stage, re-RT, ulcerative EC, esophageal stenosis, the maximum thickness of the tumor was closely related to EF. Once the EF occurs, the prognosis is extremely poor, and the conventional treatment is not effective. We ought to attach importance to the prevention of EF. We should pay attention to patients with these risk factors and choose cautious and individualized treatment methods in clinical work.

## Figures and Tables

**Figure 3:**
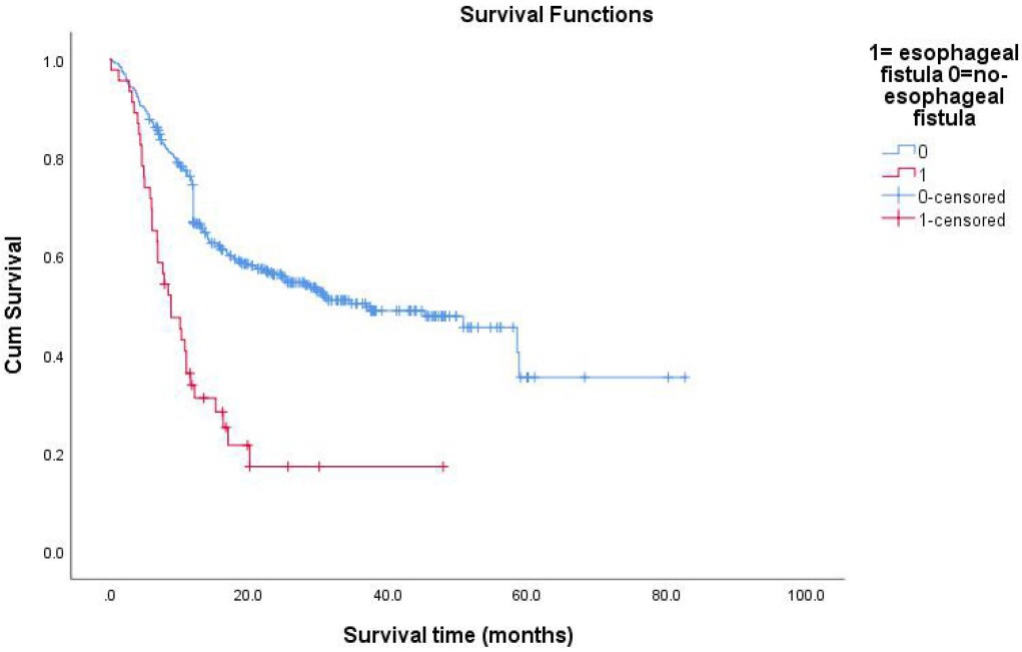
Survival functions between esophagus fistual and no-esophagus fistual.

**Figure 4:**
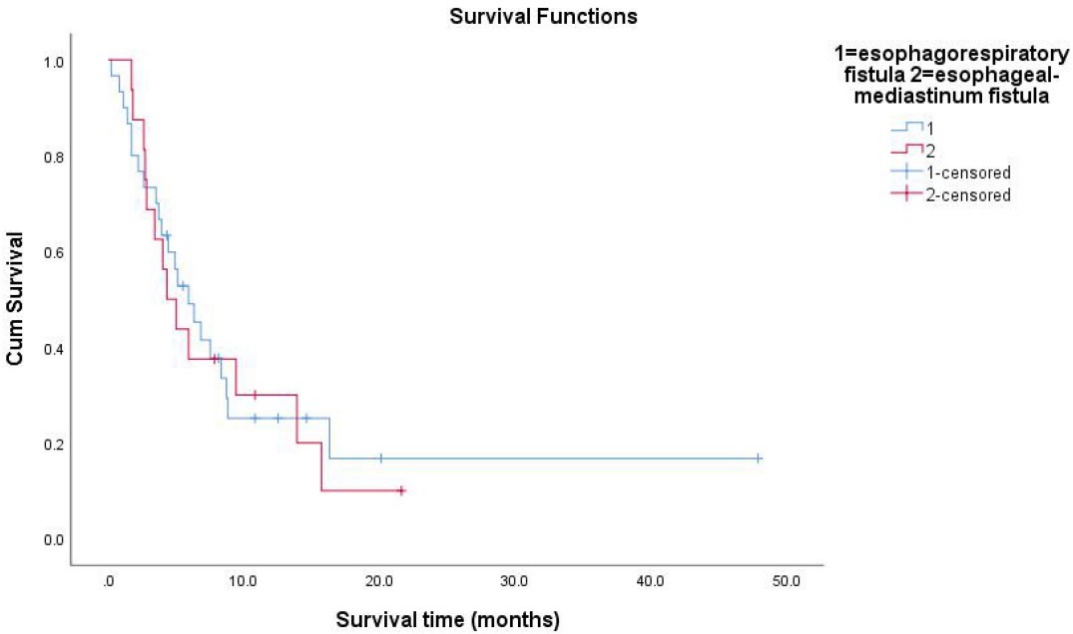
Survival functions between esophagorespiratory fistula and esophageal-mediastinum fistula.

**Table 1:** Clinical features of patients

Characteristics	Number of patients (N=414)	Number of EF patients (N=46)
**Age (years)**		
<60	112 (27.05%)	20 (43.48%)
≥60	302 (72.95%)	26 (56.52%)
**Median age (range) (years)**	65 (32~88)	60 (32~83)
**Gender**		
Female	95 (22.95%)	6(13.04%)
Male	319 (77.05%)	40(86.96%)
**KPS**		
<80	24(5.8%)	2(4.35%)
≥80	390(94.2%)	44(95.65%)
**Smoking history**		
No	200(48.3%)	18(39.13%)
Yes	214(51.69%)	28(60.87%)
**Drinking history**		
No	247(59.66%)	23(50%)
Yes	167(40.34%)	23(50%)
**T stage**		
Non-T4	259(62.56%)	19(41.3%)
T4	155(37.44%)	27(58.7%)
**N stage**		
Non- N3	389(93.96%)	39(84.78%)
N3	25(6.04%)	7(15.22%)
**M stage**		
M0	340(82.13%)	34(73.91%)
M1	74(17.87%)	12(26.09%)
**TNM clinical stage**		
IIA IIB	80(19.32%)	4(8.7%)
IIIA IIIB IIIC	259(62.56%)	31(67.39%)
IV	75(18.12%)	11(23.91%)
**Location of primary tumor**		
Cervical section	46(11.11%)	7(15.22%)
Upper thoracic	123(29.71%)	15(32.61%)
Mid thoracic	192(46.38%)	18(39.13%)
Lower thoracic	53(12.8%)	6(13.04%)
**Ulcerative tumor**		
No	260(62.8%)	21(45.65%)
Yes	154(37.2%)	25(54.35%)
**Esophageal stenosis (cm)**		
≥1	74(17.87%)	5(10.87%)
0.5-1	270(65.22%)	26(56.52%)
<0.5	70(16.91%)	15(32.61%)
**Treatment modalities**		
Without CT	98(23.67%)	6(13.04%)
Sequential CRT	196(47.34%)	24(52.17%)
Concurrent CRT	120(28.99%)	16(34.78%)
**Fraction dose (Gy)**		
<2	111(26.81%)	12(26.09%)
≥2	303(73.19%)	34(73.91%)
**Total radiation dose (Gy)**		
<60	178(43%)	16(34.78%)
≥60	236(57%)	30(65.22%)
**Re-RT**		
No	372(89.86%)	34(73.91%)
Yes	42(10.14%)	12(26.09%)
**RT field**		
IFI	139(33.57%)	14(30.43%)
ENI	275(66.43%)	32(69.57%)
**CT regimens**		
DP	191(46.14%)	30(65.22%)
PF	125(30.19%)	10(21.74%)
**Type of EF**		
EMF	16(3.86%)	16(34.78%)
ERF	30(7.25%)	30(65.22%)
AEF	0(0%)	0(0%)
**The length of primary tumor (cm)**	5.49	6.34
**Median length of tumor (range) (cm)**	5 (0.7~17.06)	7 (2~12)
**The maximum thickness of the tumor (mm)**	16.48	20.34
**Median tumor thickness (range) (mm)**	15.31 (4~41.21)	19.03 (11~38.05)

Abbreviations: AEF: Arterio-esophageal fistula; CT: chemotherapy; CRT: chemoradiotherapy; DP: docetaxel and cisplatin; EF: esophageal fistula; ENI: Elective nodal irradiation; ERF: Esophagorespiratory fistula; EMF: Esophageal-mediastinum fistula; RT: Radiotherapy; IFI: Involved field irradiation; KPS: Karnofsky performance status; PF: cisplatin and 5-fluorouracil; TNM: tumor-node-metastasis.

**Table 2:** Univariate analysis for the incidence of EF

Characteristics	EF (-)	EF(+)	OR	95% CI	P-value
**Age(years)**					
<60	92	20	1		
≥60	276	26	0.433	0.231-0.813	0.009
**Gender**					
Female	89	6	1		
Male	279	40	2.127	0.873-5.182	0.097
**KPS**					
<80	22	2	1		
≥80	346	44	0.715	0.163-3.144	0.657
**Smoking history**					
No	182	18	1		
Yes	186	28	1.522	0.814-2.848	0.189
**Drinking history**					
No	224	23	1		
Yes	144	23	1.556	0.841-2.876	0.159
**T stage**					
Non-T4	240	19	1		
T4	128	27	2.664	1.426-4.977	0.002
**N stage**					
Non- N3	350	39	1		
N3	18	7	3.49	1.372-8.878	0.009
**M stage**					
M0	306	34	1		
M1	62	12	1.742	0.854-3.552	0.127
**TNM clinical stage**					
IIA IIB	76	4	1		
IIIA IIIB IIIC	228	31	2.583	0.883-7.555	0.083
IV	64	11	3.266	0.992-10.753	0.052
**Location of primary tumor**					
Cervical section	39	7	1		
Upper thoracic	108	15	0.774	0.294-2.039	0.604
Mid thoracic	174	18	0.576	0.225-1.475	0.250
Lower thoracic	47	6	0.711	0.221-2.292	0.568
**Ulcerative tumor**					
No	239	21	1		
Yes	129	25	2.206	1.188-4.094	0.012
**Esophageal stenosis (cm)**					
<0.5	55	15	1		
0.5-1	244	26	0.391	0.194-0.786	0.008
≥1	69	5	0.266	0.091-0.776	0.015
**Treatment modalities**					
Without CT	92	6	1		
Sequential CRT	172	24	2.140	0.844-5.421	0.109
Concurrent CRT	104	16	2.359	0.886-6.281	0.086
**Fraction dose (Gy)**					
<2	99	12	1		
≥2	269	34	1.043	0.519-2.094	0.906
**Total radiation dose (Gy)**					
<60	162	16	1		
≥60	206	30	1.475	0.777-2.798	0.235
**Re-RT**					
No	338	34	1		
Yes	30	12	3.976	1.866-8.474	0.0001
**RT field**					
IFI	125	14	1		
ENI	243	32	1.139	0.585-2.219	0.702
**CT regimens**					
Non- CT	52	6	1		
DP	191	30	2.857	1.146-7.121	0.024
PF	125	10	1.333	0.467-3.805	0.591
**Cycles of CT**			1.030	0.945-1.122	0.501
**Median the length of tumor (cm)**	5	7			
**The length of tumor (range) (cm)**	(0.7-17.06)	(2-12)	1.147	1.025-1.283	0.017
**Median the maximum thickness of the tumor (mm)**	15.31	19.03			
**The maximum thickness of the tumor (range) (mm)**	(4-41.21)	(11-38.05)	1.102	1.054-1.152	0.0001

Abbreviations: CT: chemotherapy; CRT: chemoradiotherapy; CI: confidence interval; DP: docetaxel and cisplatin; EF: esophageal fistula; ENI: Elective nodal irradiation; RT: Radiotherapy; IFI: Involved field irradiation; KPS: Karnofsky performance status; OR : odds ratio; PF: cisplatin and 5-fluorouracil; TNM: tumor-node-metastasis.

**Table 3:** Multivariate analysis for the incidence of EF

Characteristics	OR	95% CI	P-value
**Age(years)**			
<60	1		
≥60	0.493	0.233-1.046	0.065
**T stage**			
Non-T4	1		
T4	2.586	1.278-5.234	0.008
**N stage**			
Non-N3	1		
N3	3.311	1.021-10.742	0.046
**Re-RT**			
No	1		
Yes	3.926	1.615-9.548	0.003
**CT regimens**			
Non- CT	1		
DP	2.056	0.717-5.895	0.180
PF	1.034	0.321-3.335	0.955
**Ulcerative tumor**			
No	1		
Yes	2.157	1.066-4.361	0.032
**Esophageal stenosis (cm)**			
<0.5	1		
0.5-1	0.345	0.149-0.795	0.012
≥1	0.260	0.078-0.865	0.028
**The length of tumor (cm)**	1.075	0.944-1.224	0.275
**The maximum thickness of the tumor (range) (mm)**	1.106	1.050-1.166	0.0001

Abbreviations: CT: chemotherapy; CI: confidence interval; CT: chemotherapy; CRT: chemoradiotherapy; DP: docetaxel and cisplatin; EF: esophageal fistula; RT: Radiotherapy; OR : odds ratio; PF: cisplatin and 5-fluorouracil.

## Abbreviations

ESCC: Esophageal squamous cell carcinoma
RT: Radiotherapy
EF: Esophageal fistula
EC: Esophageal cancer
CRT: Chemoradiotherapy
KPS: Karnofsky performance status
CT: Computed Tomography
MRI: Magnetic resonance imaging
PET-CT: Positron Emission Tomography-Computer Tomography
TXT: Docetaxel
DDP: Cisplatin
5-FU: 5-fluorouracil
GTV: Gross tumor volume
CTV: Clinical target volume
PTV: Planned target volume
OR: Odds ratio
Cis: Confidence intervals
ERF: Esophagorespiratory fistula
EMF: Esophageal-mediastinum fistula
AEF: Arterio-esophageal fistula
MST: Median survival time
